# Meckel's diverticulum perforation by a fish bone mimicking acute appendicitis: A case report and review of the literature

**DOI:** 10.1016/j.ijscr.2025.111586

**Published:** 2025-06-30

**Authors:** Sharad Bahadur Adhikari, Paribartan Baral, Namita Ranabhat, Hari Prasad Bhusal

**Affiliations:** a Tribhuvan University Teaching Hospital, Kathmandu, Nepal; b Manipal College of Medical Sciences, Pokhara, Nepal; c Kathmandu Medical College and Teaching Hospital, Pokhara, Nepal; d Gandaki Medical College Teaching Hospital, Pokhara, Nepal

**Keywords:** Meckel's diverticulum, Fish bone, Perforation, Wedge resection, Laparoscopy

## Abstract

**Introduction:**

Meckel's diverticulum (MD) is the most common congenital abnormality of the gastrointestinal tract and is usually asymptomatic. The perforation of a MD by a foreign body is a very rare complication.

**Case presentation:**

We report a 53 years old male who presented with intermittent right lower abdominal pain and tenderness in the right iliac fossa on physical examination. With a provisional diagnosis of acute appendicitis, the patient was subjected to open appendectomy. Intraoperatively perforation of MD by an intact fish bone was identified and wedge resection performed.

**Discussion:**

Meckel's diverticulum is asymptomatic in most of the individuals with symptoms occurring in only 4–16 %. The clinical presentation ranges from intestinal obstruction, to bleeding, inflammation and perforation. Perforation of a MD due to a foreign body is extremely rare event and most cases are diagnosed during the operation. The treatment of symptomatic MD is surgical resection.

**Conclusion:**

Perforation of MD should be kept in mind as a differential diagnosis in the evaluation and management of abdominal pain.

## Highlights

• Meckel's diverticulum is generally asymptomatic but sometimes may present with symptoms and complications.
• Perforation of MD by a fishbone is a very rare occurrence but should be kept in mind during evaluation of abdominal pain.
• Symptomatic MD requires surgical resection either by open or laparoscopic methods.
• Laparoscopic excision is currently the modality of treatment for symptomatic MD.

## 1 Introduction

Meckel's diverticulum is the most common congenital anomaly of the gastrointestinal tract which is caused by the incomplete obliteration of the omphalomesenteric duct during fifth to seventh weeks of gestation occurring in 2 to 4 % of the population [1]. A ‘rule of two’ applies to the MD: occurring in 2 % of the population, length of about 2 inches., located within 2 feet proximal to the ileocecal valve, found in children under 2 years of age and affects males twice as often as females [2].

Most patients with MD are asymptomatic, but when symptomatic children usually present with gastrointestinal bleeding and adults with intestinal obstruction. Perforation of the MD by foreign bodies is a very rare complication because they pass through the gastrointestinal tract without significant consequences [3]. We present an unusual case of perforation of MD by an intact fish bone mimicking as acute appendicitis which was diagnosed during the surgery. This case report has been reported in line with the SCARE Criteria [4].

## 2 Case presentation

A 53 years old gentleman presented to the Emergency Department with complaints of intermittent right lower abdominal pain of 2 days which was increasing in severity since the past 6 hours. The pain initially started from the periumbilical region later on localizing to the right iliac fossa and associated with nausea. However, there was no associated history of vomiting, fever and any urinary complaints. He did not have any significant past medical and surgical history.

Initial abdominal examination revealed both tenderness and rebound tenderness in the Right Iliac fossa along with positive Rovsing's sign. Examination of the respiratory and cardiovascular system was normal. Laboratory examination revealed increased white blood cell count of 14,000 (4000–11,000/μl) with neutrophil count of 81 %.

Imaging with ultrasonography of the abdomen revealed non visualization of the appendix with minimal interloop bowel fluid. The patient was managed in the ED with intravenous fluids, antibiotics and analgesics. The patient was subjected to emergency open appendectomy because of the unavailability of routine laparoscopic services in the hospital.

**Fig. 1:**
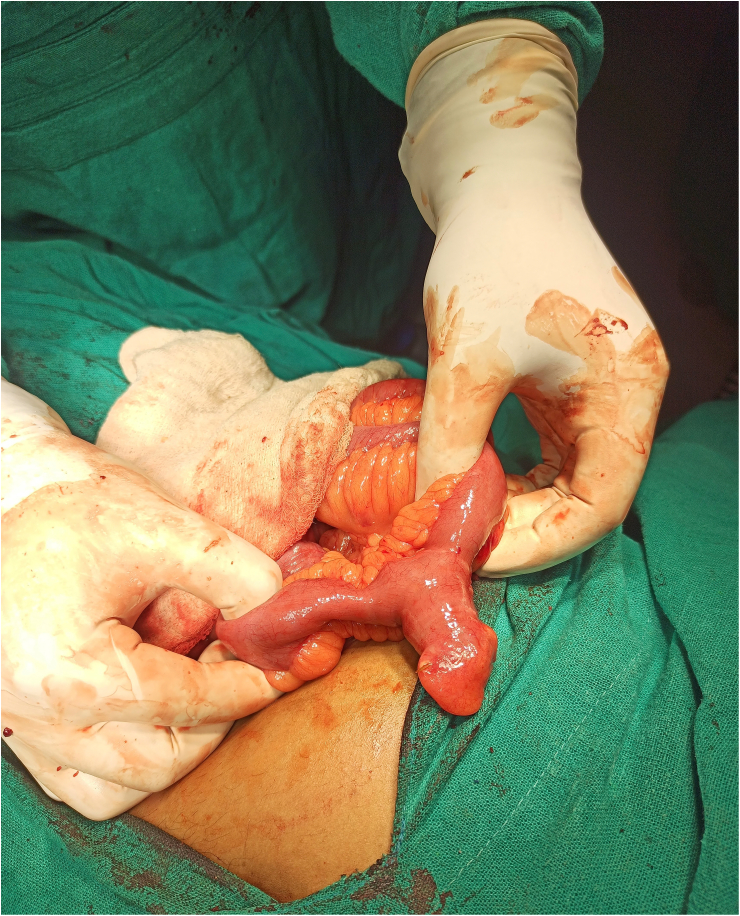
Intraoperative demonstration of Meckel's diverticulum.

**Fig. 2:**
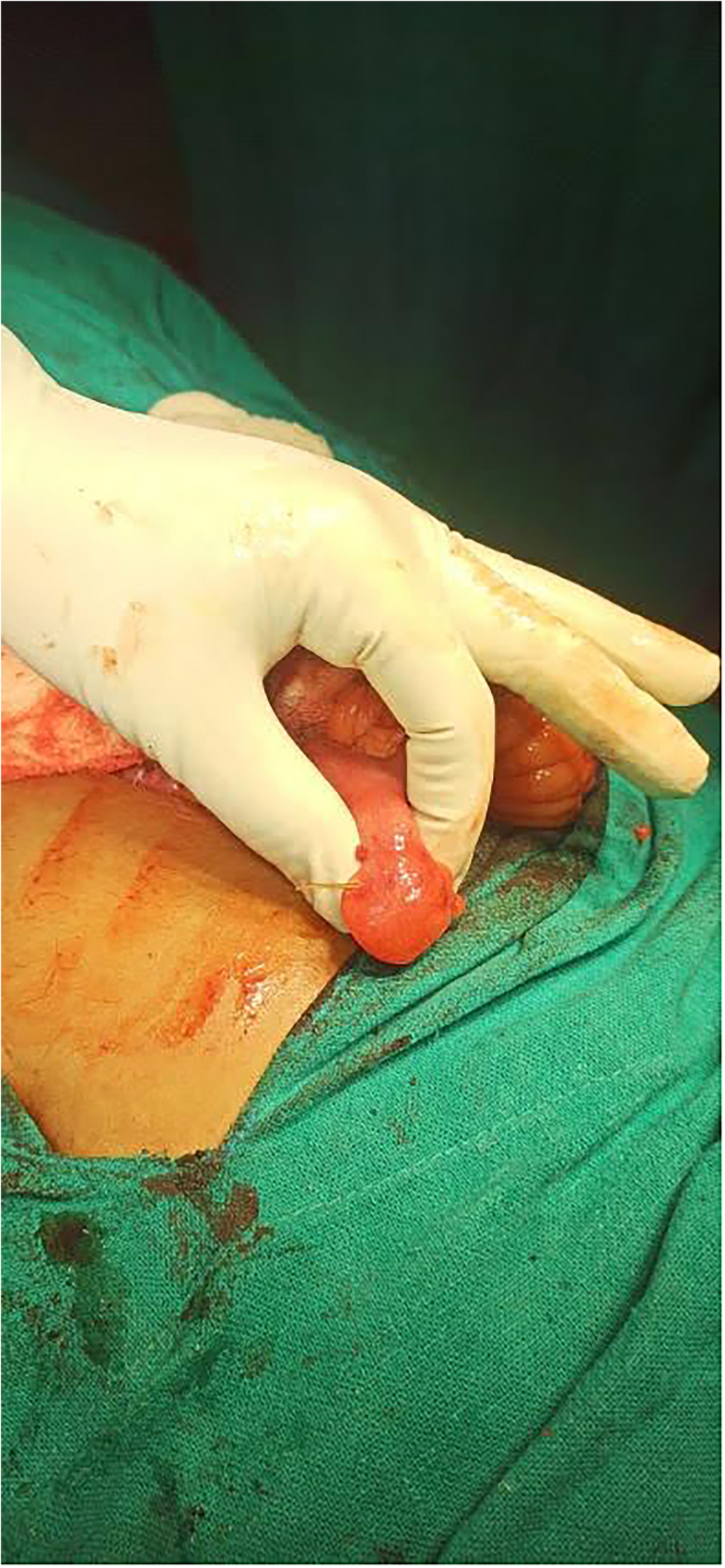
Demonstration of fish bone protruding through the Meckel's diverticulum.

**Fig. 3:**
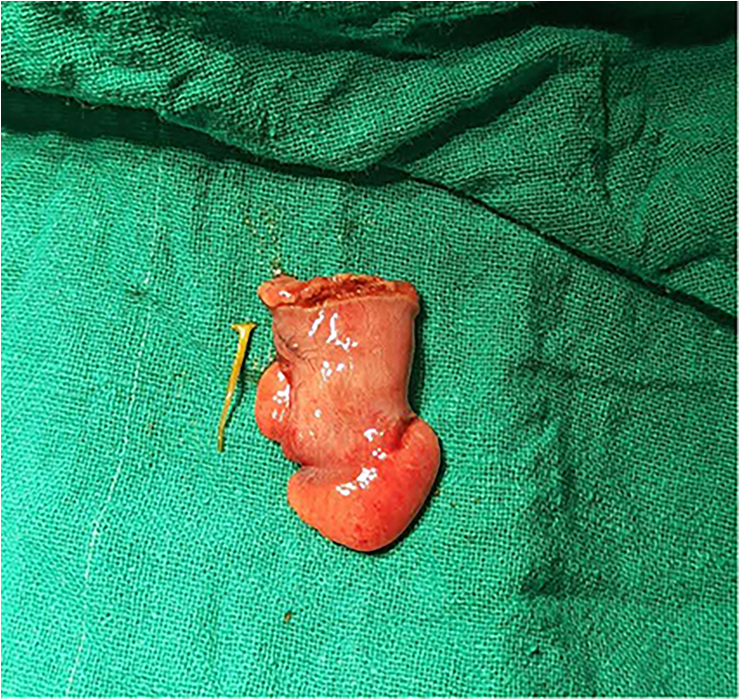
Resected Meckel's diverticulum along with the fish bone (note the inflamed tip).

During the surgery appendix was found to be completely normal and healthy following which thorough examination of the small bowel was done which revealed an inflamed and perforated Meckel's diverticulum due to an impacted intact fish bone approximately 80 cm proximal to ileocecal junction. Wedge resection of the Meckel's diverticulum and transverse suturing of the ileal defect along with an appendectomy was performedThe specimen was subjected to histopathological examination which revealed inflamed Meckel's diverticulum and no evidence of ectopic tissue or malignancy. On follow up after 6 months he is healthy without any issues (Fig. 1, Fig. 2, Fig. 3).

## 3 Discussion

Meckel's diverticulum was first described by a German surgeon Fabricius Hildanus in 1598 and named by Johann Friedrich Meckel who explained the anatomy and embryology of MD [5]. It consists of a small outpouching of the gastrointestinal tract due the incomplete obliteration of the omphalomesenteric duct between the 5th to 7th weeks of fetal life [6]. It is a true diverticulum containing all layers of the intestinal wall and its own blood supply from the superior mesenteric artery, which makes it vulnerable to obstruction and infection [7].

Most patients are asymptomatic, with only 4–16 % presenting with symptoms such as inflammation, haemorrhage, and intestinal obstruction. Other complications reported are diverticulitis, perforation and enterolith formation [1,2].

When symptoms occur, it is commonly caused by ingestion of foreign bodies and multiple foreign bodies have been identified as a cause of MD perforation, with fish bones accounting for 55 % of those cases [8]. The majority of ingested foreign bodies do not cause significant symptoms as they pass easily through the gastrointestinal tract and only 1 % will require surgery [9]. Ward‐McGuard. suggested the mechanism of the perforation to be a combination of local inflammation due to irritation of the foreign body, and pressure necrosis of the divertculum wall, due to attempts by peristalsis to push the foreign body toward the tip of the diverticulum [18].

Quoting the line of Charles Mayo “Meckel's diverticulum is frequently suspected, often looked for, but seldom found” [1], only less than 10 % of symptomatic Meckel's diverticula are diagnosed preoperatively [10], and acute appendicitis is the most common preoperative diagnosis as in our case [6]. As majority of the patients do not recall an episode of fish bone ingestion and may present non-specific signs and symptoms including abdominal pain, vomiting, intestinal obstruction and gastrointestinal bleeding, clinical diagnosis may become challenging [8,11]. However upon retrospective questioning our patient recalled having a course of fish meal 3 days earlier.

Plain radiographs and abdominal ultrasonography may be normal or show nonspecific changes and CT scan of uncomplicated MD can resemble a normal bowel loop. However, during a case of fishbone impaction or perforation, there may be certain changes in the CT scan like thickened intestinal wall, localized pneumoperitoneum, fatty deposits, intraabdominal abscess and a linear hypodense structure in the abdominal cavity surrounded by inflammatory changes [12].

The approach to treatment of a MD depends on whether it is symptomatic or an incidental finding [13]. There is a considerable debate on the management of incidental Meckel's diverticulum. Robijn et al. devised a risk score weighted on male sex, age under 45 years, length over 2 cm and presence of a fibrous band [14]. The risk score has not yet been validated although frequently mentioned by several studies and authors.

It is generally agreed that resection should be undertaken in case of symptomatic Meckel's diverticulum [10]. The surgical approach could be via laparotomy, laparoscopy or laparoscopy assisted procedures. The surgical procedure depends on intraoperative findings and complications and could be a simple diverticulectomy with wedge resection or segmental ileal resection with end-to-end anastomosis [15]. Laparoscopy has recently emerged as a minimally invasive approach and is currently the modality of choice in cases of different abdominal pathologies including MD. The advent of gastrointestinal stapling devices has made excision faster, safer and more efficient. One of the important advantage of stapling is that it closes the bowel lumen as it cuts, which completely reduces the chance of peritoneal contamination [16]. A simple wedge resection of the diverticulum and closure of the ileal defect would suffice in a narrow-base MD without any palpable mass in the lumen [17]. Suturing of the ileal defect should be done in a transverse fashion to avoid narrowing the ileal lumen [10] Indications of bowel resection include wide mouth MD with ectopic tissue, bleeding MD, inflamed and ischemic adjacent ileum, inflamed or perforated base and involvement of the MD by tumors [7]. In cases of gross contamination of the peritoneal cavity, segmental bowel resection and fashioning a double barrel ileostomy may be rarely needed.

This case report reminds the clinicians to consider pathology of Meckel's diverticulum in patients with abdominal pain even in adult patients as the diverticulum might be asymptomatic until any pathology results in the inflammation and symptoms like in our case. Also, this case report highlights the need of thorough exploration of the bowel to rule out other abdominal pathology if we encounter normal appendix in the setting of surgery for suspected acute appendicitis.

## 4 Conclusion

The perforation of a Meckel diverticulum by a foreign body like fishbone is a rare event and should be considered in the differential diagnosis of acute abdominal pain with atypical symptoms. It is most commonly diagnosed preoperatively as acute appendicitis and careful exploration of the bowel should be done intraoperatively in cases of normal appendix. Symptomatic MD should undergo resection and laparoscopy has been found to be an effective diagnostic and therapeutic tool.

## CRediT authorship contribution statement

1. Dr Sharad Bahadur Adhikari Tribhuvan University Teaching Hospital: Study concept, primary surgeon, paper writing, editing the final manuscript
2. Dr Paribartan Baral: Study concept, editing the final manuscript, assistant surgeon
3. Dr Namita Ranabhat: Literature search, data collection, writing manuscript
4. .Dr Hari Prasad Bhusal: Obtaining consent, data collection, literature search.

## Consent

Written informed consent was obtained from the patient for publication of this case report and accompanying images. A copy of the written consent is available for review by the Editor-in-Chief of this journal on request.

## Ethical approval

Ethical approval is exempt at our institute.

## Guarantor

Dr Sharad Bahadur Adhikari.

## Funding

None.

## Declaration of competing interest

All authors declare that they do not have any conflicts of interest.
